# Immunoexpression of proliferation and apoptosis markers in oral vascular anomalies

**DOI:** 10.1590/0103-6440202205010

**Published:** 2022-12-05

**Authors:** Tiago João da Silva, Denise Hélen Imaculada Pereira de Oliveira, Cassiano Francisco Weege Nonaka, Éricka Janine Dantas da Silveira, Lélia Maria Guedes Queiroz

**Affiliations:** 1 School of Dentistry, State University of Paraíba, Campina Grande, PB, Brazil.; 2 Postgraduate Program Health Sciences, Federal University of Ceara, Sobral, CE, Brazil.; 3 Postgraduate Program, Dentistry, State University of Paraíba, Campina Grande, PB, Brazil.; 4 Postgraduate Program in Oral Pathology, Department of Dentistry, Federal University of Rio Grande do Norte, Natal, RN, Brazil.

**Keywords:** hemangioma, vascular anomalies, pathology, cell proliferation, apoptosis

## Abstract

The biological behavior of lesions is highly dependent on the imbalance between their proliferative and apoptotic capacity. This study evaluated a correlation between the proliferative and apoptotic rates of different oral vascular anomalies (VAs) by analyzing the immunoexpression of proliferation (Ki-67) and apoptosis (Bcl-2 and Bax) markers in endothelial cells of 20 cases of GLUT-1 positive infantile hemangiomas (IHs), 20 cases of pyogenic granulomas (PGs) and 20 cases of vascular malformations (VMs). Immunoexpression analysis of Ki-67, Bcl-2 and Bax revealed a lower median percentage of positive cells in VMs cases compared to IHs and PGs cases (*P* <0.001). The Wilcoxon signed‐rank test showed significantly higher percentages of immunostaining for Bax than for Bcl‐2 in IHs (*P* = 0.048). In the group of PGs, a positive correlation was observed between the immunoexpressions of Ki-67 and Bax (*r* = 0.476; *P* = 0.034). Although oral IHs, PGs and VMs present similar clinical and histopathological features, each of these lesions has its etiopathogenic particularities. The results of this study suggest that different biological behaviors of VAs may be related to differences in the proliferative and apoptotic profiles of their endothelial cells.

## Resumo

O comportamento biológico das lesões é altamente dependente do desequilíbrio entre sua capacidade proliferativa e apoptótica. Neste estudo, avaliou-se se existe correlação entre as taxas proliferativas e apoptóticas de diferentes anomalias vasculares orais (AVs) analisando a imunoexpressão de marcadores de proliferação (Ki-67) e apoptose (Bcl-2 e Bax) em células endoteliais de 20 casos de hemangiomas infantis GLUT-1 positivos (HIs), 20 casos de granulomas piogênicos (GPs) e 20 casos de malformações vasculares (MVs). A análise de imunoexpressão de Ki-67, Bcl-2 e Bax revelou uma porcentagem mediana menor de células positivas nos casos de MVs em comparação aos casos de HIs e GPs (P <0,001). O teste de Wilcoxon mostrou porcentagens significativamente maiores de imunocoloração para Bax do que para Bcl-2 em HIs (P = 0,048). No grupo dos GPs, foi observada correlação positiva entre as imunoexpressões de Ki-67 e Bax (r = 0,476; P = 0,034). Embora HIs, GPs e MVs orais apresentem características clínicas e histopatológicas muito semelhantes, cada uma dessas lesões tem suas particularidades etiopatogênicas. Os resultados deste estudo sugerem que diferentes comportamentos biológicos dos AVs podem estar relacionados a diferenças nos perfis proliferativo e apoptótico de suas células endoteliais.

## Introduction

The term “vascular anomalies” (VA) comprises a wide range of pathologies with similar clinical and histological features, including vascular malformations (VMs) and proliferative vascular tumors. The vascular tumors group includes neoplastic lesions, such as infantile hemangiomas (IH), and non-neoplastic reactive lesions, such as pyogenic granulomas (PG) ^1^ ^-^ ^8^.

VMs are the result of errors in vasculogenesis, which do not exhibit cell proliferative activity. In fact, the blood vessels that accumulate in these lesions gradually increase in diameter without proliferation of the vascular endothelium cells ^3^ ^,^ ^4^ ^,^ ^7^ ^,^ ^9^. PGs are non-neoplastic proliferative lesions, with rapid potential growth, and histopathologically characterized by the presence of inflammation and angiogenesis ^7^ ^,^ ^8^ ^,^ ^10^. Conversely, IHs are true neoplastic lesions characterized by increased expression of proangiogenic factors, such as fibroblast growth factor (FGF) and vascular endothelial growth factor (VEGF), proliferation of endothelial cells, and remodeling of extracellular matrix. Nonetheless, at some point of IHs development, angiogenesis is downregulated, mesenchymal cells start to differentiate into adipocytes and endothelial cells undergo apoptosis ^1^ ^,^ ^3^ ^,^ ^4^ ^,^ ^5^ ^,^ ^7^ ^,^ ^11^.

Even though oral IHs, PGs and VMs have similar clinical and histopathological features, each of these three different lesions has its etiopathogenic particularities. Therefore, this study aimed to evaluate the immunoexpression of proliferation (Ki-67) and apoptosis (Bcl-2 and Bax) markers in 20 IHs, 20 PGs and 20 VMs of the oral cavity, already revised by immunoexpression of GLUT-1 and histopathological features ^5^ ^,^ ^7^ ^,^ ^12^, in order to better understand the biological behavior and etiopathogenesis of these lesions.

## Materials and methods

Twenty IHs, 20 PGs and 20 VMs were used. All specimens were previously analyzed by their GLUT-1 immunopositivity and it was considered as true IHs only the cases that showed positivity staining for the GLUT-1 marker. All PG and VM cases were GLUT-1 negative and were diagnosed according to their different morphological aspects as it was shown in a previous study performed by our group ^5^. Specimens with other associated lesions and cases previously submitted to any therapy were excluded.

We submitted the formalin-fixed paraffin-embedded tissues samples to hematoxylin-eosin stain and immunohistochemistry using anti-Ki-67 (MIB-1, Dako, Carpinteria, CA, USA), anti-Bcl-2 (124, Dako, Carpinteria, CA, USA) and anti-Bax (E63, Abcam, Cambrigde, MA, USA) antibodies. For all antibodies, tonsillar tissue sections were used as positive control. Negative control consisted of bovine serum albumin as replacement for the primary antibodies. The expression of the Ki-67 marker was analyzed by the positive staining in the nucleus of the endothelial cells. For Bcl-2 and Bax all endothelial cells that exhibited nuclear and/or cytoplasmic brown staining were classified as positive and the endothelial cells that showed complete absence of staining were classified as negative. The immunoexpression of Ki-67 was evaluated quantitatively, whereas semi-quantitative analysis was performed for Bcl-2 and Bax (score 0: 0-5%; score 1: 6-50%; score 2: 51-100%) (13]). The results were analyzed statistically using the nonparametric Kruskal-Wallis, Wilcoxon signed-rank and Spearman correlation tests, with a 5% level of significance.

## Results

**Figure1:**
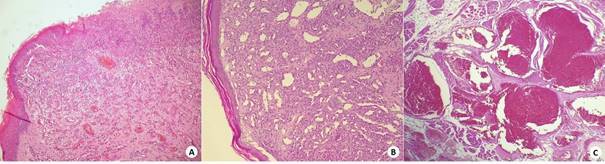
. Photomicrograph showing histopathological characteristics (Hematoxylin / Eosin) of the VAs present in the current study: (A) PG, (B) HI, (C) VM. (ADVANCE; 400x).

**Figure 2:**
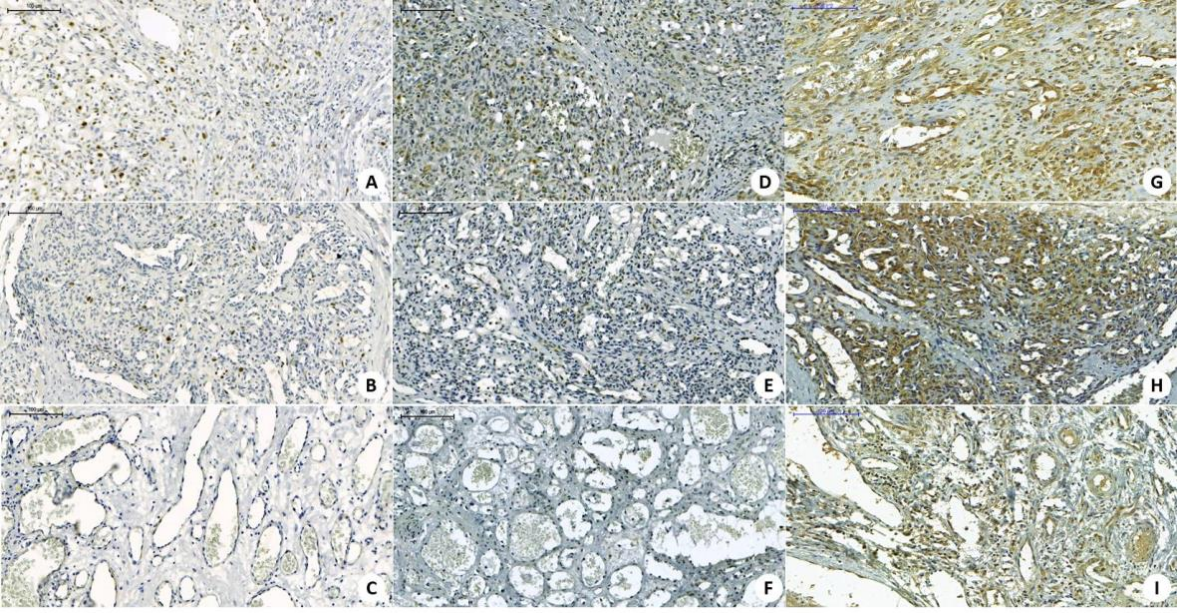
Photomicrography showing an Immunoexpression of Ki-67 in PG (A), IH (B) and VM (C); Immunoexpression of Bcl-2 in PG (D), IH (E) and VM (F); Bax immunoexpression in PG (G), IH (H) and VM (I) - Panoramic viewer 1.15.2 (3DHISTECH® Kft. 29-33, Konkoly-Thege M. str. Budapest, Hungary, H-1121).

Clinicopathological characteristics, such as sex, age, location, size and evolution time are summarized in Table 1 and the morphological characteristics in Figure 1. Analysis of the immunoexpression of Ki-67 revealed a significant difference among groups with a lower median percentage of positive cells in cases of VM (4.5%) compared to IH (13.8%) and PG (33.7%) (*P* < 0.001) (Table 2) (shown in Fig. 2A - 2C). Regarding the immunoexpression of Bcl-2 and Bax, VMs showed lower median percentage of positive cells compared to IHs and PGs (*P* < 0.001) (Table 2) (shown in Fig. 2D - 2I). The Wilcoxon signed‐rank test showed significantly higher percentages of immunostaining for Bax than for Bcl‐2 in IHs (*P* = 0.048) (Table 3). In the group of PGs, a positive correlation was observed between the immunoexpressions of Ki-67 and Bax (*r* = 0.476; *P* = 0.034).

**Table 1:** Distribution of the frequency of sex, age, location, size and evolution time of oral VAs.

Vascular anomalies			
	PG	IH	VM
SEX (n)			
Female	13	11	12
Male	7	9	8
AGE (years old)			
Minimum	11	8	24
Maximum	73	81	80
LOCATION (n)			
Gingiva/ Ridge	2	5	1
Lip	7	5	7
Palate	1	3	3
Buccal mucoa	3	3	5
Tongue	1	2	1
Others	4	2	1
Missing	2	0	2
SIZE (cm)			
< 1	11	8	8
1 - 3	2	4	3
> 3	2	1	1
Missing	5	7	8
EVOLUTION TIME			
Minimum	1 week	4 weeks	1 month
Maximum	4 years	3 years	5 years

PG: Pyogenic granuloma; IH: Infantile hemangioma; VM: Vascular malformation.

**Table 2:** Sample size, median, quartiles 25 and 75, mean rank, KW statistic and statistical significance for Ki-67, Bcl-2 and Bax immunoexpression according to type of VA.

	Lesion	n	Median	Q_25_-Q_75_	Mean rank	KW	*P*
Ki-67	IH	20	13.8	6.5 - 19.9	29.60	28.807	<0.001
	PG	20	33.7	25.2 - 50.6	45.75		
	VM	20	4.5	2.6 - 11.0	16.15		
Bcl-2	IH	20	1.0	0.0 - 2.0	33.10	17.803	<0.001
	PG	20	1.5	1.0 - 2.0	39.85		
	VM	20	0.0	0.0 - 0.0	18.55		
Bax	IH	20	2.0	1.0 - 2.0	33.80	17.148	<0.001
	PG	20	2.0	2.0 - 2.0	38.80		
	VM	20	0.0	0.0 - 1.0	18.90		

**Table 3:** Distribution of cases [n (%)] of IH, PG and VM according to ranks of percentage of immunopositive cells for Bcl-2 and Bax.

Lesion	Bcl-2 > Bax	Bcl-2 < Bax	Bcl-2 = Bax	*P*
IH	3 (15.0)	9 (45.0)	8 (40.0)	0.048
PG	3 (15.0)	8 (40.0)	9 (45.0)	0.154
VM	4 (20.0)	7 (35.0)	9 (45.0)	0.118

## Discussion

It is widely known that the biological behavior of any lesion is highly dependent on the balance between the levels of cell proliferation and cell death, and that some immunohistochemical markers, such as Ki-67, Bcl-2 and Bax may help to identify the dimension of these aspects. In this study we found a higher expression of Ki-67, Bcl-2 and Bax in PG when compared to the other VAs. This high proliferation activity observed in PG cases may be explained by their reactional, inflammatory nature ^3^ ^,^ ^8^ ^,^ ^14^. In this context, trauma or poor oral hygiene stimulates the recruitment of inflammatory cells, which release cytokines that induce angiogenesis and cell proliferation, followed by an impaired wound healing process that brings forth more growth factors, such as VEGF, FGF and transforming growth factor alpha (TGF-α) ^8^ ^,^ ^14^ ^,^ ^15^ ^,^ ^16^. PGs are not well characterized from a standpoint of cell death so far, but the higher level of Bcl-2 immunostaining observed in this study suggests suppression of apoptosis, which was also found in a previous study ^17^. This condition may be associated to the rapid growth of these tumors, which reinforces the concept that lower levels of apoptosis may favor tumor growth, as proposed by Wu et al. ^18^ Indeed, it was expected a lower expression of Bax, the proapototic marker, which was not observed in the PG cases studied. Nakamura et al. ^17^ suggested that Bcl-2 family proteins contribute to the suppression of apoptosis in PG, at least in part. Nevertheless, the role of Bax in this imbalance between proliferation and apoptosis in VAs is still difficult to speculate.

In the present study, it was evaluated only true IHs cases that were previously GLUT-1 tested ^5^ as proposed by North et al. ^12^. IHs are characterized by a proliferative phase, followed by a spontaneous regression phase and then an involuted phase, which is characterized by a final balance with few remaining capillary-like vessels surrounded by loose fibrofatty tissue ^3^ ^,^ ^7^ ^,^ ^19^. Most of the specimens of IH evaluated in this study were in the involuted phase, as shown in our previous publications ^5^ ^,^ ^8^ ^,^ ^20^. Ki-67 evaluation showed lower levels of positivity in comparison to PGs and higher levels when compared with VMs. Also, it was observed higher immunopositivity of Bax than Bcl-2 marker in the IH specimens. These findings support the fact that even though IHs are true benign neoplasms, they present this particularity of having an involutive phase characterized by endothelial apoptosis and the downregulation of angiogenesis. However, the precise molecular mechanisms of regression are still unknown ^3^ ^,^ ^7^ ^,^ ^19^.

VMs are lesions that exhibit normal endothelial turnover ^3^ ^,^ ^7^. Some studies affirmed that VMs do not have hyperplastic endothelial cells ^9^ ^,^ ^21^ ^,^ ^22^, although they may eventually demonstrate expansion due to stimuli such as trauma or infection ^23^_._ In the VMs analyzed it was observed a minimum proliferative rate by Ki-67 immunoexpression. Similar findings were also detected by Meier-Jorna et al. ^13^ which found endothelial proliferative activity in 30% of the skin VMs analyzed and by Osaki et al. ^24^ which found Ki-67 positivity in some cases of orbital VMs (≤1%). The lower rates of Bcl-2 and Bax were found in the VM specimens suggesting that these proteins do not affect them. Information regarding the immunoexpression of Bcl-2 family of proteins in VMs is scarce. In a study with VMs of central nervous system, Takagi et al. ^25^ suggested that cell death by apoptosis plays a role in the development and maintenance of these lesions.

The results of this study suggest that different biological behaviors of VAs could be related to differences in proliferative and apoptotic profiles. Taken together, these findings reinforce the importance of the correct diagnosis and classification of these lesions, based on ISSVA (2018), for a better understanding of their different clinical progression.
